# Knowledge and Awareness on Orofacial Clefts Among Healthcare Students: A Cross‐Sectional Study

**DOI:** 10.1155/ijod/3757733

**Published:** 2025-12-15

**Authors:** Kanza A. Rasyid, Asty S. Setiawan, Fidya M. Putri

**Affiliations:** ^1^ Undergraduate Study Program, Faculty of Dentistry, Padjadjaran University, Bandung, Indonesia, unpad.ac.id; ^2^ Department of Dental Public Health, Faculty of Dentistry, Padjadjaran University, Bandung, Indonesia, unpad.ac.id

**Keywords:** awareness, health students, knowledge, orofacial clefts

## Abstract

**Introduction:**

Orofacial clefts require multidisciplinary management and if not properly intervened can impair various functions and reduce the quality of life of the patient. Good knowledge about orofacial clefts is very important for health students to prepare for comprehensive case management. The purpose of this study was to determine the level of knowledge and awareness of Padjadjaran University health students regarding orofacial clefts.

**Methods:**

This study used a cross‐sectional method with a survey study, where the measuring instrument used was a questionnaire given online to undergraduate students of dentistry, medicine, midwifery, and nursing at Padjadjaran University with a total population of 2611 students. The sample was taken by stratified random sampling using the binomial proportion sample size formula with a minimum of 315 respondents from all study programs. The questionnaire was self‐developed with 16 questions in the knowledge section and eight questions in the awareness section. This questionnaire underwent validity and reliability testing, with results exceeding the correlation coefficient value (0.361) and Cronbach’s *α* (0.891). Data were analyzed by frequency distribution with categorization of knowledge and awareness levels, and the chi‐square test with CI 95% and *α* = 0.05.

**Results:**

Respondents were predominantly female (89.2%), aged 20–22 years (65.4%), and from urban areas (62.2%). The majority (52.7%) of students had good knowledge of orofacial clefts, with dental students showing the best knowledge. A total of 77.5% of respondents had good awareness, with dentistry’s the highest (97.6%). Knowledge and awareness increased with age and level of study. Students who had attended orofacial clefts lectures showed better knowledge and awareness than those who had not.

**Conclusion:**

The majority of health students had good knowledge and awareness of the orofacial clefts, increasing with semester and related course experience, with variation between courses showing differences in curriculum emphasis.

## 1. Introduction

Orofacial clefts are one of the most common congenital anomalies worldwide, with a prevalence of one in 700 births globally [1, 2]. This condition can manifest as a cleft lip, cleft palate, or a combination of both, resulting from a complex interaction between genetic and environmental factors [3]. Indonesia reports a prevalence of congenital anomalies in children of 59.3 per 1000 live births, with Jakarta having the highest rate for orofacial clefts at 13.9 per 1000 births [4]. The physiological difficulties caused by orofacial clefts can impair speech, language, eating, and other developmental processes [1]. Untreated orofacial clefts can lead to death [3]. The management of orofacial clefts is crucial given their impact on the quality of life of affected individuals [5]. Individuals with orofacial clefts face significant physical and psychological stress, including aesthetic concerns, difficulties with breastfeeding and feeding, which can lead to nutritional issues. Managing this condition requires a multidisciplinary team comprising pediatricians, dentists, plastic surgeons, nutritionists, and speech therapists [6–9].

Awareness of orofacial clefts among healthcare professionals and the community remains low, especially in remote areas, rural areas, and among the poor [3]. Parents’ and community awareness of orofacial clefts is still lacking, making it crucial for health students to understand the diagnosis, treatment, and education of orofacial clefts to increase public awareness [5, 10]. Self‐awareness also plays an important role in how individuals assess their knowledge, which affects confidence in providing healthcare services [11]. The relationship between knowledge and awareness of health students regarding orofacial clefts is very close and significant, where knowledge provides the foundation for understanding etiology, symptoms, and disease prevention, while awareness reflects the ability to recognize the importance of this information in clinical contexts [12].

Early detection through antenatal examination and prenatal ultrasonography has been proven to improve treatment prognosis and quality of life for patients [5, 13]. This detection process requires comprehensive pregnancy examination and counseling involving midwives, nurses, and obstetrician–gynecologist specialists [13]. Ultrasound during the second trimester of pregnancy allows for early detection of orofacial clefts, providing time for parents to prepare [14]. Diagnosis of orofacial clefts still faces challenges due to insufficient standard protocols, especially for cleft palate. Ultrasonographic examination may encounter obstacles due to factors such as gestational age, maternal obesity, amniotic fluid volume, accompanying fetal abnormalities, fetal movement, or the examining physician’s experience [15]. Diagnosis of isolated cleft palate is often difficult to perform before birth and is frequently missed during the neonatal period. Nonoptimal detection is attributed to insufficient thorough palatal examination and minimal knowledge regarding orofacial clefts [16].

Research shows that the level of knowledge and awareness among health students regarding orofacial clefts remains limited. A study conducted on final‐year medical students in England demonstrated that more than half of the students still had doubts about their level of knowledge regarding cleft lip and palate [17]. Research on dental students in Peru showed that the majority had poor knowledge about the management of patients with cleft lip and palate [18]. Research in Vietnam found that the majority of dental and medical students showed adequate levels of awareness regarding orofacial clefts [19]. Research about orofacial clefts knowledge in health students in Indonesia remains very limited, with only one study conducted at the Universitas Sumatra Utara that focused exclusively on dental students as research subjects [20]. There is no research about the level of knowledge and awareness among health students not only for dentistry or medicine students regarding orofacial clefts in Indonesia. This study aims to determine the level of knowledge and awareness of health students at Padjadjaran University regarding orofacial clefts.

## 2. Materials and Methods

This study is a descriptive observational study with a cross‐sectional approach. Primary data collection was conducted through online questionnaires using the Google Forms platform, from February to March 2025. This study has been approved by the Research Ethics Committee of Padjadjaran University with Reference Number 1249/UN6.KEP/EC/2024.

The population in this study consists of undergraduate students from dentistry, medicine, midwifery, and nursing programs at Padjadjaran University. The sampling technique used was stratified random sampling. The sample size was calculated using the cross‐sectional study formula with a binomial proportion with confidence level 95% and *z*‐score 1.96. The total population of this study was 2611 students across all programs that consist of medicine: 1055 students, dentistry: 539 students, midwifery: 142, nursing: 875 students; with a minimum sample size determined to be 315 respondents [21]. The minimum sample size required for the dentistry program was 82 respondents, medicine 88 respondents, midwifery 58 respondents, and nursing 87 respondents.

The research instrument consisted of a questionnaire in Bahasa Indonesia with two sections. The first section measured respondents’ knowledge with 16 questions covering aspects of etiology, epidemiology, prevalence, detection methods, impacts, and treatment procedures for orofacial clefts. The second section assessed respondents’ awareness with eight questions about understanding prevalence, impacts, early detection, management of orofacial clefts, and the role of healthcare professionals. Validity and reliability tests were conducted to avoid bias in this study. Validity was tested through face validity (interviews with five respondents from each study program) assessed the clarity in term of language aspect, the clarity in term of easiness to answer, feasibility to access the online form, design or display of the online form, and duration to fill the form. Content validity was conducted by four experts (Department of Pedodontics, Oro‐maxillofacial Surgery, Oral Biology, and Dental Public Health), and Pearson product moment (involving 30 respondents) with results greater than the table value (0.361). The reliability test results using Cronbach’s *α* was 0.891. Data collection was conducted through individual contact with respondents listed in the sampling frame that had been established.

The analysis results are presented based on knowledge and awareness categories with three levels: good, adequate, and poor. Knowledge was measured using the Guttman scale (true, false) and categorized as good at 76%–100%, fair at 56%–75%, and poor if the percentage was below 56% [22]. Awareness was measured using a Likert scale (strongly disagree, disagree, undecided, agree, strongly agree) with categorization of good at 80%–100%, fair at 60%–79%, and poor if the percentage was below 60% [23]. This study uses descriptive statistics such as frequency distribution analysis in terms of numbers and percentages, and the results are presented using tables. The chi‐square test (*α* = 0.05 and confidence level 95%) used to analyze the association between the characteristics (study program, academic semester, gender, residence, and history of attending the lecture) with level of knowledge and awareness. The questionnaire data was processed using IBM SPSS Version 29.0.2.0 and Microsoft Excel Version 16.102.2.

## 3. Result

**Figure 1:**
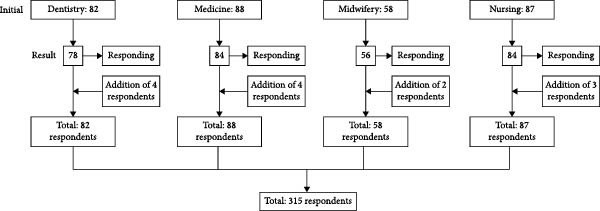
Respondent distribution flow.

This study involved respondents from four health study programs with a recruitment flow that shows the initial population, response rate, and addition of respondents to obtain the minimum sample required for this study, the flow is shown as follows (Figure 1).

The total number of respondents was 315, the target sample size was achieved with a response rate of 100%. The results of the descriptive analysis based on the data in the research tables are detailed as follows.

Table 1 shows the results of the frequency distribution based on respondent characteristics. The respondent frequency distribution results show a dominance of females (89.2%), aged 20–22 years (65.4%), and from municipality areas (62.2%). The study program proportions are evenly distributed, with the majority of respondents being 7th–8th semester students (28.3%). A total of 47.9% of respondents have attended orofacial cleft lectures.

**Table 1:** Frequency distribution based on respondent characteristics.

Respondent characteristics	Frequency (*n*)	Percentage (%)
Gender		
Male Female	34 281	10.8 89.2
Age (years)		
17–19 20–22 23 or older	105 206 4	33.3 65.4 1.3
Residence		
Municipality Regency	196 119	62.2 37.8
Undergraduate study program		
Dentistry Medicine Midwifery Nursing	82 88 58 87	26 27.9 18.4 27.6
Academic semester		
Semester 1–2 Semester 3–4 Semester 5–6 Semester 7–8 Semester 9 or above	70 60 96 89 —	22.2 19 30.5 28.3 —
History of attended lectures on orofacial clefts or related topics		
Already attended Not yet attended Not sure Don’t know	151 84 52 28	47.9 26.7 16.5 8.9

The highest percentage of correct answers for Table 2 was found in Question 2 regarding the location of orofacial clefts, with 286 respondents (90.8%). Table 2 shows that respondents had fairly good knowledge of orofacial clefts. Significant gaps in knowledge were observed in several questions. The majority of respondents (53.7%) answered Question 5 incorrectly regarding the prevalence of cleft lip. The greatest misunderstanding was found in the primary goal of initial treatment (Question 14), with 57.8% of respondents answering incorrectly.

**Table 2:** Frequency distribution of knowledge section questionnaire responses.

Questionnaire items	Correct		Incorrect	
	*n*	%	*n*	%
1. Orofacial clefts are congenital or birth defects.	277	87.9	38	12.1
2. Orofacial clefts can occur in the lip, palate, or both.	286	90.8	29	9.2
3. Cleft lip cannot occur bilaterally.	222	70.5	93	29.5
4. Orofacial clefts are formed due to growth disturbances in the fusion between the palate and lip of the fetus starting from the fourth to ninth week of fetal age.	243	77.1	72	22.9
5. Cleft lip defects occur more frequently in females than males.	146	46.3	169	53.7
6. A combination of genetic and environmental factors is one of the causes of orofacial clefts.	247	784	68	21.6
7. Smoking and alcohol consumption during pregnancy can increase the risk of orofacial clefts in children.	270	85.7	45	14.3
8. Folic acid deficiency during pregnancy poses a risk for orofacial clefts.	258	81.9	57	18.1
9. Babies with orofacial clefts do not experience difficulty breastfeeding.	240	76.2	75	23.8
10. Individuals with cleft palate suffer from breathing disorders (sleep apnea).	238	75.6	77	24.4
11. Orofacial clefts can be detected at 18 weeks of pregnancy using ultrasonography (USG).	195	61.9	120	38.1
12. The team involved in treating babies born with orofacial cleft conditions requires multidisciplinary care including pediatricians, pediatric dentists, orthodontists, and craniofacial surgeons.	261	82.9	54	17.1
13. Individuals with orofacial clefts do not require treatment from speech therapists.	256	81.3	59	18.7
14. The main goal of initial treatment for babies with cleft palate is to improve speech ability.	133	42.2	182	57.8
15. Palatoplasty is one of the surgical procedures to close cleft palate.	245	77.8	70	22.2
16. Patients with cleft lip and palate can be directly breastfed without requiring assistive devices for food intake.	225	71.4	90	28.6

*Note: n*, frequency; %, percentage.

Table 3 shows the distribution of questionnaire responses in the awareness section. The analysis results indicate a good level of awareness regarding orofacial clefts. The statement regarding the role of healthcare professionals in providing education to the community about orofacial clefts received the highest percentage, with 225 respondents (71.4%) stating “strongly agree”. A total of 51.1% of respondents answered “strongly agree” to having heard about orofacial clefts; however, only 26.7% answered “strongly agree” that this condition is the most commonly encountered disorder.

**Table 3:** Frequency distribution of awareness section questionnaire responses.

Questionnaire items	SD	D	N	A	SA
	*n* (%)	*n* (%)	*n* (%)	*n* (%)	*n* (%)
1. I have heard about orofacial clefts (cleft lip and/or cleft palate).	2 (0.6)	29 (9.2)	36 (11.4)	87 (27.6)	161 (51.1)
2. I am aware that orofacial clefts are one of the most common facial and oral cavity abnormalities.	—	32 (10.2)	82 (26)	117 (37.1)	84 (26.7)
3. I understand that orofacial clefts can affect the quality of life of those who have them.	—	28 (8.9)	28 (8.9)	89 (28.3)	170 (54)
4. I understand that orofacial clefts in babies can cause problems with speaking and eating.	—	26 (8.3)	27 (8.6)	82 (26)	180 (57.1)
5. I am aware that orofacial clefts can affect the psychosocial well‐being of children who suffer from them.	1 (0.3)	27 (8.6)	25 (7.9)	87 (27.6)	175 (55.6)
6. I understand the importance of early diagnosis and management of orofacial clefts.	10 (3.2)	23 (7.3)	26 (8.3)	68 (21.6)	188 (59.7)
7. I am aware that the treatment of orofacial clefts requires a multidisciplinary approach (involving various specialists).	1 (0.3)	12 (3.8)	41 (13)	74 (23.5)	187 (59.4)
8. I consider the role of healthcare professionals important in providing education to the community about orofacial clefts.	—	4 (1.3)	16 (5.1)	70 (22.2)	225 (71.4)

Abbreviations: A, agree; D, disagree; N, neutral; SA, strongly agree; SD, strongly disagree.

Table 4 shows that dental students have the best level of knowledge about orofacial clefts. Medical students show an almost equal distribution between the good and fair categories. The midwifery study program has the highest level of poor knowledge (17.2%) among all study programs. The majority (52.7%) of health students have good knowledge about orofacial clefts. The statisticts test shown the *p*‐value = 0.022 (*p*  < 0.05).

**Table 4:** Frequency distribution of health students’ knowledge level regarding orofacial clefts based on study program.

Study program	Knowledge level category						*p*‐Value ^∗^
	Good		Fair		Poor		
	*n*	%	*n*	%	*n*	%	
Dentistry	55	67.1	22	26.8	5	6.1	—
Medicine	37	42	38	43.2	13	14.8	0.022 ^∗∗^
Midwifery	26	44.8	22	38	10	17.2	—
Nursing	48	55.2	25	28.7	14	16.1	—

Total (% out of 315)	166	52.7	107	34	42	13.3	—

*Note: n*, frequency; %, percentage.

^∗^Chi‐square test.

^∗∗^ *p*  < 0.05.

Table 5 shows that dental students have the highest level of awareness (97.6%) among all study programs. The midwifery program has the highest proportion of respondents with poor awareness category (17.2%). The majority of respondents (77.5%) have good awareness about orofacial clefts, but there are still 10.2% of students with poor awareness category. The statisticts test shown the *p*‐value = 0.001(*p*  < 0.05).

**Table 5:** Frequency distribution of health students’ awareness level regarding orofacial clefts based on study program.

Study program	Awareness level category						*p*‐Value ^∗^
	Good		Fair		Poor		
	*N*	%	*n*	%	*n*	%	
Dentistry	80	97.6	2	2.4	—	—	—
Medicine	58	65.9	18	20.5	12	13.6	0.001 ^∗∗^
Midwifery	38	65.6	10	17.2	10	17.2	—
Nursing	68	78.2	9	10.3	10	11.5	—

Total (% out of 315)	244	77.5	39	12.3	32	10.2	—

*Note: n*, frequency; %, percentage.

^∗^Chi‐square test.

^∗∗^ *p*  < 0.05.

Table 6 shows that the category of good knowledge is relatively balanced between male (55.9%) and female (52.3%). Knowledge increases with age, with the ≥ 23 age group showing the highest level of good knowledge (75%). All study programs show an increase in knowledge as the level of study increases, except for the nursing study program, which shows a decrease at the fourth level. Students who have taken the orofacial cleft course have significantly better knowledge than those who have not. The medical program achieved the highest percentage (82.1%) of good knowledge after taking the course. The highest level of uncertainty was found among nursing students (68.4%), with 37.5% stating they were unaware of the orofacial cleft course. The chi‐square test shown that for all study programs, age, academic semester, and history of attended lectures associated with the level of knowledge (*p*  < 0.05), except the gender (*p*  > 0.05).

**Table 6:** Frequency distribution of health students’ knowledge level regarding orofacial clefts based on gender, age, academic level, and exposure to orofacial cleft topics.

Respondent characteristics	Good		Fair		Poor		*p*‐Value ^∗^
	*n*	%	*n*	%	*n*	%	
Gender							
Male Female	19 147	55.9 52.3	10 97	29.4 34.5	5 37	14.7 13.2	0.835
Age (years)							
17–19 20–22 23 or older	23 140 3	21.9 68 75	50 56 1	47.6 27.2 25	32 10 —	30.5 4.9 —	0.001 ^∗∗^
Dentistry							
Academic semester							
Semester 1–2 Semester 3–4 Semester 5–6 Semester 7–8	3 9 13 30	25 64.3 56.5 90.9	5 4 10 3	41.7 28.6 43.5 9.1	4 1 — —	33.3 7.1 — —	0.001 ^∗∗^
History of attended lectures on orofacial clefts or related topics							
Already attended Not yet attended Not sure Don’t know	51 2 2 —	75 25 33.3 —	15 3 4 —	22.1 37.5 66.7 —	2 3 — —	2.9 37.5 — —	0.001 ^∗∗^
Medicine							
Academic semester							
Semester 1–2 Semester 3–4 Semester 5–6 Semester 7–8	— 10 23 4	— 37 65.7 66.7	14 13 9 2	70 48.1 25.7 33.3	6 4 3 —	30 14.8 8.6 —	0.001 ^∗∗^
History of attended lectures on orofacial clefts or related topics							
Already attended Not yet attended Not sure Don’t know	23 6 8 —	82.1 18.2 47.1 —	4 17 8 9	14.3 51.5 47.1 90	1 10 1 1	3.6 30.3 5.9 10	0.001 ^∗∗^
Midwifery							
Academic semester							
Semester 1–2 Semester 3–4 Semester 5–6 Semester 7–8	3 2 9 12	15.8 25 56.3 80	8 6 6 2	42.1 75 37.5 13.3	8 — 1 1	42.1 — 6.3 6.7	0.001 ^∗∗^
History of attended lectures on orofacial clefts or related topics							
Already attended Not yet attended Not sure Don’t know	15 7 4 —	75 38.9 40 —	5 7 3 7	25 38.9 30 70	— 4 3 3	— 22.2 30 30	0.006 ^∗∗^
Nursing							
Academic semester							
Semester 1–2 Semester 3–4 Semester 5–6 Semester 7–8	3 5 20 20	15.8 45.5 90.9 57.1	7 5 2 11	36.8 45.5 9.1 31.4	9 1 — 4	47.4 9.1 — 11.4	0.001 ^∗∗^
History of attended lectures on orofacial clefts or related topics							
Already attended Not yet attended Not sure Don’t know	28 4 13 3	80 16 68.4 37.5	7 14 4 —	20 56 21.1 —	— 7 2 5	— 28 10.5 62.5	0.001 ^∗∗^

*Note: n*, frequency; %, percentage.

^∗^Chi‐square test.

^∗∗^ *p*  < 0.05.

Table 7 shows that awareness levels increase with age, with the ≥ 23 age group achieving a perfect awareness level (100%). The dentistry program maintained a good awareness category throughout all semesters (91.3%–100%) with no instances of poor awareness. The nursing and midwifery programs show a gradual increase, while the medical program experiences a decrease in Semesters 7–8. Students who have taken courses on orofacial clefts have a high level of awareness, while those who have never taken such courses show a high level of poor awareness (50%). The chi‐square test shown that for all study program, the gender is not associated with the awareness level, meanwhile the age is associated. Each study program has different result from the statistics test. All the study program, except dentistry, show results that academic semester and history of attended lectures that related with the orofacial cleft topics are assosiated with the awareness level.

**Table 7:** Frequency distribution of health students’ awareness level regarding orofacial clefts based on gender, age, academic level, and exposure to orofacial cleft topics.

Respondent characteristics	Good		Fair		Poor		*p*‐Value ^∗^
	*n*	%	*n*	%	*n*	%	
Gender							
Male Female	26 218	76.5 77.6	7 32	20.6 11.4	1 31	2.9 11	0.133
Age (years)							
17–19 20–22 23 or older	57 183 4	54.3 88.8 100	18 21 —	17.1 10.2 —	30 2 —	28.6 1 —	0.001 ^∗∗^
Dentistry							
Academic semester							
Semester 1–2 (*n* = 12) Semester 3–4 (*n* = 14) Semester 5–6 (*n* = 23) Semester 7–8 (*n* = 33)	12 14 21 33	100 100 91.3 100	— — 2 —	— — 8.7 —	— — — —	— — — —	0.154
History of attended lectures on orofacial clefts or related topics							
Already attended Not yet attended Not sure Don’t know	66 8 6 —	97.1 100 100 —	2 — — —	2.9 — — —	— — — —	— — — —	0.810
Medicine							
Academic semester							
Semester 1–2 (*n* = 20) Semester 3–4 (*n* = 27) Semester 5–6 (*n* = 35) Semester 7–8 (*n* = 6)	7 17 30 4	35 63 85.7 66.7	4 7 5 2	20 25.9 14.3 33.3	9 3 — —	45 11.1 — —	0.001 ^∗∗^
History of attended lectures on orofacial clefts or related topics							
Already attended Not yet attended Not sure Don’t know	25 20 12 1	89.3 60.6 70.6 10	3 6 4 5	10.7 18.2 23.5 50	— 7 1 4	— 21.2 5.9 40	0.001 ^∗∗^
Midwifery							
Academic semester							
Semester 1–2 (*n* = 19) Semester 3–4 (*n* = 8) Semester 5–6 (*n* = 16) Semester 7–8 (*n* = 15)	6 6 13 13	31.6 75 81.3 86.7	4 1 3 2	21.1 12.5 18.8 13.3	9 1 — —	47.4 12.5 — —	0.002 ^∗∗^
History of attended lectures on orofacial clefts or related topics							
Already attended Not yet attended Not sure Don’t know	17 11 8 2	85 61.1 80 20	3 5 1 1	15 27.8 10 10	— 2 1 7	— 11.1 10 70	0.001 ^∗∗^
Nursing							
Academic semester							
Semester 1–2 (*n* = 19) Semester 3–4 (*n* = 11) Semester 5–6 (*n* = 22) Semester 7–8 (*n* = 35)	7 8 20 33	36.8 72.7 90.9 94.3	4 1 2 2	21.1 9.1 9.1 5.7	8 2 — —	42.1 18.2 — —	0.001 ^∗∗^
History of attended lectures on orofacial clefts or related topics							
Already attended Not yet attended Not sure Don’t know	34 16 16 2	97.1 64 84.2 25	1 4 2 2	2.9 16 10.5 25	— 5 1 4	— 20 5.3 50	0.001 ^∗∗^

*Note: n*, frequency; %, percentage.

^∗^Chi‐square test.

^∗∗^ *p*  < 0.05.

## 4. Discussion

Research on health students’ knowledge and awareness of orofacial clefts provides important insights into the readiness of future healthcare professionals in dealing with congenital cases. The results of this study show a dominance of female respondents, which reflects the tendency of health professions to be of greater interest to women. This finding is consistent with the World Health Organization (WHO) statement that 70% of healthcare workers worldwide are dominated by women [24]. The majority of respondents were aged 20–22, indicating that most students are in the middle of their studies, which may influence their level of understanding. A person’s age influences one’s way of thinking and cognitive abilities, according to Darsini et al. [25], as age increases, thinking abilities improve, leading to the development of knowledge.

The demographic analysis results of participants show a relatively even distribution across health study programs. Respondents were dominated by urban students, indicating inequities in access or opportunities for higher education, which aligns with findings by Lestari et al. [26], regarding the preference of rural youth who prefer working over attending university. The diversity of study programs provides a multidisciplinary perspective on understanding orofacial clefts as a condition requiring integrated management from various health professionals [27]. Participation in lectures about orofacial clefts shows varied results, indicating the possibility of curriculum differences between health study programs regarding this topic [28–31].

The research results show variations in the level of knowledge and awareness among health students regarding orofacial clefts. The study by Agha et al. [5] was consistent with the results of this study, which showed that knowledge about the location of orofacial cleft occurrence received the highest percentage of correct answers. The high number of correct answers for this question is due to orofacial clefts topic being included in the curriculum across all study programs [28–31].

Knowledge gaps are evident in questions regarding cleft lip prevalence, consistent with research by Babu and Rakshagan [32], that dental students’ knowledge about cleft lip prevalence is quite poor. Students generally misunderstand that complete cleft lip occurs more frequently in males while isolated cleft palate is more commonly found in females [33]. Many respondents also misunderstood the main purpose of initial treatment for babies with cleft palate, contrary to the statement by Phalke et al. [27] that the goal of orofacial clefts treatment is to address breathing problems and feeding issues.

The role of healthcare workers in educating the public about orofacial clefts shows the highest level of awareness. This finding is consistent with the study conducted by Neto et al. [34], which found that nursing students in Brazil were fully aware of the importance of their role and the need to educate the community, especially pregnant women. Competency guidelines from the Ministry of Health emphasize the importance of patient communication and education as core competencies in health education programs. The healthcare professional competencies established by the Ministry of Health prioritize the dissemination of health knowledge to the community as preventive action efforts [35]. Awareness about orofacial clefts as the most commonly encountered facial and oral cavity abnormalities is relatively low. This fact indicates the need for increased education regarding the prevalence of this condition.

The research results show that the majority of health students have good knowledge about orofacial clefts, with dental students dominating the highest knowledge level. This finding is not consistent with research by Babu and Rakshagan [32], who found that dental students’ knowledge was quite lacking and did not meet standards. The insufficient knowledge level was attributed to a lack of exposure to certain materials related to orofacial clefts. The difference from this study is also due to variations in the curriculum emphasis [29, 32].

The majority of medical students have a good level of knowledge about orofacial clefts, in contrast to the findings of Rehman et al. [17], which showed that more than half of medical students have a low level of knowledge about cleft lip and palate. This low level of knowledge is attributed to the fact that most of the respondents who participated in the study had not been exposed to materials related to orofacial clefts, were not involved in the care of such cases, and lacked confidence in their current research conducted by Picinato et al. shows that healthcare professionals and health students including nursing, medicine, and dentistry have a fairly good level of knowledge. These findings indicate that the utilization of websites as additional information sources effectively improves health students’ knowledge regarding orofacial clefts. Consistent with these research results, healthcare students have good knowledge about orofacial clefts [36]. Research on the level of knowledge regarding orofacial clefts among midwifery students is still limited, with no studies found that use samples from this study program.

The majority of health students have a good level of awareness about orofacial clefts, indicating adequate understanding and a positive picture of future healthcare professionals’ readiness in identifying and managing these cases. This finding is consistent with research by Le Kha et al. [19], who found a good level of awareness, but dental students had better comprehend compared to medical students at Hanoi Medical University. The more specific focus on the orofacial field in dental education is the reason why dental students show stronger interest, enthusiasm, and desire to learn compared to medical students. Studies on awareness of orofacial clefts among nursing and midwifery students are still very limited, and none have specifically evaluated these two programs.

The knowledge level of health students is relatively balanced between male and female respondents despite the unbalanced number of respondents. The equivalent percentage of good knowledge indicates that gender is not a determining factor in understanding orofacial clefts. This result contradicts research by Agha et al., showing that female respondents had higher knowledge levels compared to male respondents on certain topics such as CLP prevalence and related oronasal complications, which are more related to theoretical aspects. The teaching methods applied can influence student performance. Female respondents’ mastery of theoretical aspects tends to show superior performance compared to male respondents [5]. Darsini et al. [25] stated that age affects thinking ability and comprehension, with knowledge increasing as age increases. The results of this study support this statement, showing that respondents’ knowledge increases with age.

All study programs showed a gradual increase in knowledge based on the level of study. Third‐year students demonstrated superior knowledge compared to second‐year students, and second‐year students exhibited better knowledge than first‐year students. This finding is consistent with Arthanari’s study, which reported that higher academic levels correlate with improved knowledge of orofacial clefts [37]. Arthanari’s opinion is supported by research conducted by Guerra–Carrillo et al. [38], which shows that higher education levels will result in better knowledge levels. This knowledge improvement indicates that the integrating education system successfully helps students understand the material better as their time in university increases.

The nursing study program showed a different pattern, with the highest level of knowledge at the third level and decrease at the fourth level. This decrease in knowledge level is supported by findings from Narnaware et al. [39], which explains that long exposure time to material can affect a person’s memory regarding the topic that has been given. Nursing students in this study were exposed to material on orofacial clefts in their second year of study, resulting in a decline in their knowledge levels by the fourth year due to the lack of repetition of this topic over the following 2 years [31].

Students with exposure to orofacial cleft topic have much better knowledge levels compared to those who have not been exposed, are unsure, or do not know whether they have received such topic. The results of this study are consistent with research conducted by Kamilah, that someone who receives information will also gain more knowledge about that topic [40]. Exposure to orofacial cleft topic in the learning process proves to be a determining factor in forming good understanding among students regarding orofacial clefts.

Research by Kusumaningrum and Dewi [41] shows significant differences in awareness between female and male. Female generally have higher levels of awareness compared to male. This study shows no significant difference in awareness levels between the two genders [41]. Students’ awareness levels regarding orofacial clefts also show an improvement pattern based on age. The results of this study are consistent with research conducted by Wettstein et al. [42], that a person’s awareness will increase with age. Increased awareness is also influenced by factors such as life experience, education level, and cognitive development that occurs throughout a person’s lifespan [42].

The level of awareness among health students tends to increase as they progress through their semesters and are exposed to lecture topic on orofacial clefts. Variations between semesters and study programs indicate differences in the emphasis of topics in the curriculum between study programs. The effectiveness of learning is evident from the high level of awareness among students who have attended these lectures. The research by Le Kha et al. [19] supports these findings, showing that medical and dental students have very good awareness with increased self‐confidence as the level of lectures progresses. The increasingly comprehensive and in‐depth lecture topic at higher levels of study helps students understand orofacial clefts gradually and more effectively.

This study had limitations regarding difficulties in contacting a small proportion of respondents from the predetermined sampling frame, thus researchers utilized an additional sampling frame to ensure adequate sample size achievement according to established calculations. The use of online forms such as google forms for this research has limited control over who respond. Confirmation by sampling frame, contacted by the researcher to ensure that the student get the information and filled the form. Multiple submission also could happened, routine verification and handling the duplicate to ensure the data conducted during the data collection. Compared to previous studies, this research not only measuring knowledge levels but also evaluating respondents’ awareness of orofacial clefts, as well as involves a more representative sample from various health student programs that include orofacial clefts topic in their curriculum.

## 5. Conclusion

Based on research of the knowledge and awareness levels of health students about orofacial clefts at Padjadjaran University, it can be concluded that the majority of health students have good levels of knowledge and awareness regarding orofacial clefts. Students’ knowledge and awareness levels tend to increase with advancing semesters and lecture experience about orofacial clefts, with variations among health study programs showing differences in material emphasis within the curriculum at Padjadjaran University.

## Conflicts of Interest

The authors declare no conflicts of interest.

## Author Contributions

Conceptualization: **Fidya M. Putri**. Methodology: **Fidya M. Putri** and **Asty S. Setiawan**. Data collection, validation, and draft writing: **Kanza A. Rasyid**. Supervision, review and editing of the manuscript: **Fidya M. Putr** **i** and **Asty S. Setiawan**. Each author participated equally in the data analysis.

## Funding

This study was not supported by any external funding.

## Data Availability

The datasets used in this study are available from the corresponding author upon reasonable request and with appropriate justification.
